# Surface radiation dose comparison of a dedicated extremity cone beam computed tomography (CBCT) device and a multidetector computed tomography (MDCT) machine in pediatric ankle and wrist phantoms

**DOI:** 10.1371/journal.pone.0178747

**Published:** 2017-06-01

**Authors:** Sebastian Tschauner, Robert Marterer, Eszter Nagy, Georg Apfaltrer, Michael Riccabona, Georg Singer, Georg Stücklschweiger, Helmuth Guss, Erich Sorantin

**Affiliations:** 1 Division of Pediatric Radiology, Department of Radiology, Medical University of Graz, Graz, Austria; 2 Division of General Pediatric and Adolescence Surgery, Medical University of Graz, Graz, Austria; 3 Competence Centre for Medical Physics and Radiation Protection, University Hospital Graz, Graz, Austria; Leibniz Institute for Prvention Research and Epidemiology BIPS, GERMANY

## Abstract

**Objectives:**

To evaluate and compare surface doses of a cone beam computed tomography (CBCT) and a multidetector computed tomography (MDCT) device in pediatric ankle and wrist phantoms.

**Methods:**

Thermoluminescent dosimeters (TLD) were used to measure and compare surface doses between CBCT and MDCT in a left ankle and a right wrist pediatric phantom. In both modalities adapted pediatric dose protocols were utilized to achieve realistic imaging conditions. All measurements were repeated three times to prove test-retest reliability. Additionally, objective and subjective image quality parameters were assessed.

**Results:**

Average surface doses were 3.8 ±2.1 mGy for the ankle, and 2.2 ±1.3 mGy for the wrist in CBCT. The corresponding surface doses in optimized MDCT were 4.5 ±1.3 mGy for the ankle, and 3.4 ±0.7 mGy for the wrist. Overall, mean surface dose was significantly lower in CBCT (3.0 ±1.9 mGy vs. 3.9 ±1.2 mGy, p<0.001). Subjectively rated general image quality was not significantly different between the study protocols (p = 0.421), whereas objectively measured image quality parameters were in favor of CBCT (p<0.001).

**Conclusions:**

Adapted extremity CBCT imaging protocols have the potential to fall below optimized pediatric ankle and wrist MDCT doses at comparable image qualities. These possible dose savings warrant further development and research in pediatric extremity CBCT applications.

## Background

Extremity cone beam computed tomography (CBCT) represents an imaging modality that produces cross-sectional studies from volumetric acquisitions. It employs an X-ray tube and a flat-panel detector rotating opposite to each other in a gantry [1–3]. The main difference to modern multidetector computed tomography (MDCT) scanners operated in volumetric mode is the use of a flat panel detector and the absence of pre-detector collimation [2, 3]. The resulting simplicity of the CBCT construction facilitates small and mobile machines [4] and weight-bearing applications [5–11].

CBCT as an imaging method is not a new concept, and it is widely used in dental imaging for instance [12–18]. Previous studies on extremity CBCT indicated comparable diagnostic value compared to MDCT and characterized it as a possible alternative in adult orthopedic imaging [19–22]. Recently, initial experiences of pediatric CBCT applications were published as well [23].

Extraordinary dose-saving potentials in adults have been described in the knee and ankle region [24, 25]. These low-dose capabilities drew attention of pediatric radiology research on the new modality lately. Pugmire et al. published lower extremity CBCT doses in children compared to MDCT [23]. Conversely, Lang et al. and Neubauer et al. did not substantiate these findings and reported comparable doses of CBCT and MDCT examinations, when taking image quality into account [20, 22]. As a consequence, actual CBCT dose savings compared to MDCT remain a legitimate subject for debate and research.

**Fig 1:**
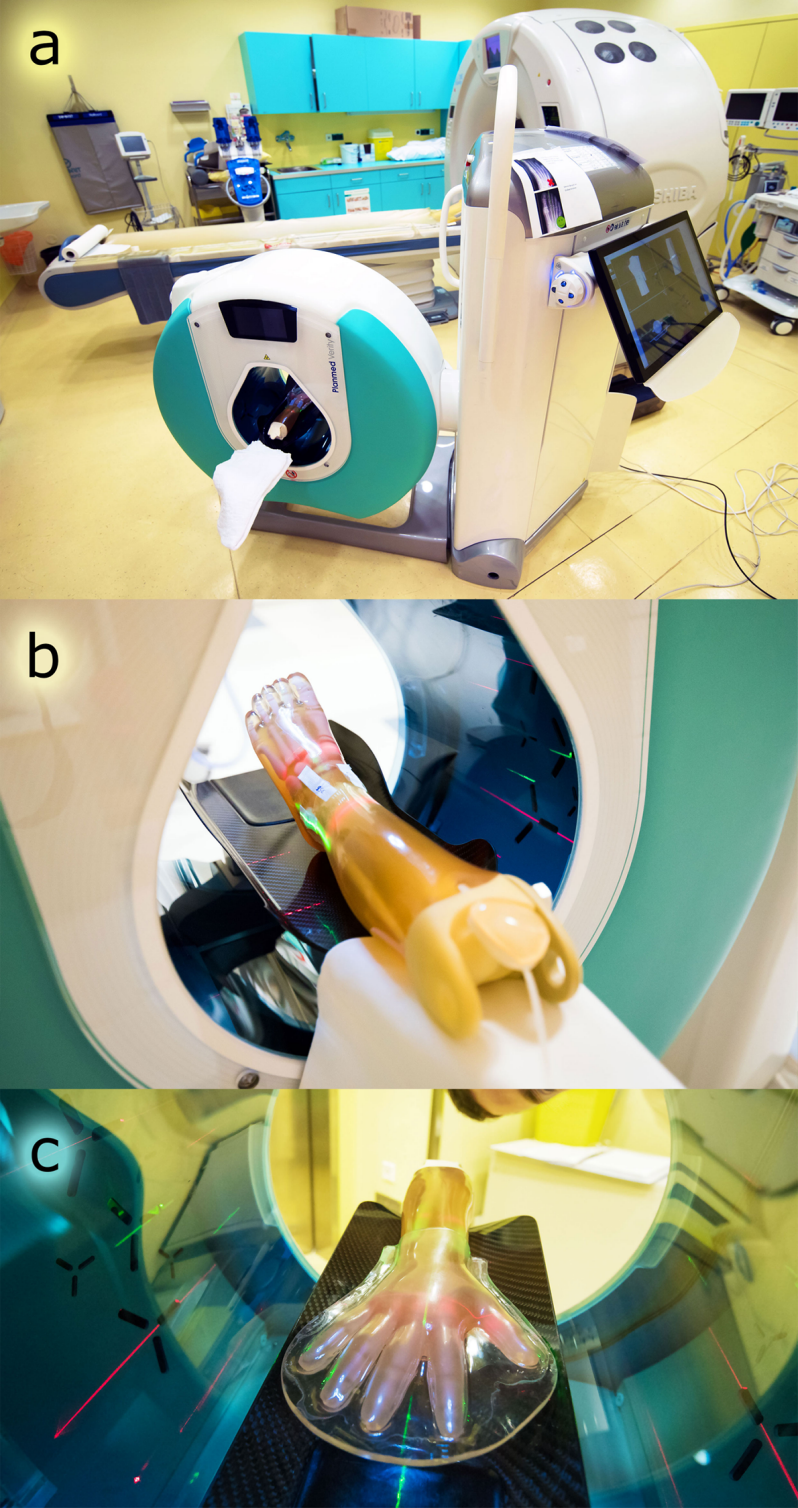
Images of measurement setup. a) CBCT (front) and MDCT (background) scanners used for dosimetry, b) pediatric ankle phantom in the CBCT gantry, c) pediatric wrist phantom in the CBCT gantry.

The purpose of the current study was to compare surface radiation doses of an established and optimized MDCT and a novel extremity CBCT scanner, both depicted in Fig 1a, in pediatric anthropomorphic ankle and wrist phantoms. Adapted pediatric imaging protocols on both machines were used to achieve realistic results at our dedicated pediatric radiology division. Surface doses were assessed by employing thermoluminescence dosimeters (TLDs) [26].

## Methods

Dosimetric studies were conducted on a left lower leg and a right forearm of a pediatric whole body phantom PBU-70 (Kyoto Kagaku Co. Ltd, Kyoto, Japan) [27]. The anthropomorphic phantoms were modelled after a 4-year-old child of 105 cm height and 20 kg weight. The used extremities embedded cortical and cancellous bone in soft tissue equivalent material (Fig 1b and 1c).

CBCT dosimetry was performed on a Planmed Verity^®^ scanner (Planmed Oy, Helsinki, Finland). Aided by laser position markers, the phantoms were centered in the middle of the gantry. Both extremities were positioned proximo-distally with the intra-articular spaces of either the talocrural or the radioulnar joint at the center (compare Fig 1b and 1c). The field of view (FOV) had a diameter of 16 cm and an extension of 12 cm proximo-distally. CBCT tube rotation angle was 210°.

Correspondingly, multidetector computed tomography (MDCT) dosimetric studies were conducted on a Toshiba Aquilion One^®^ (Toshiba Medical Systems Corporation, Otawara-shi, Japan), a 320-row scanner. Equally to the CBCT, ankle and wrist phantoms were centered in the gantry. Proximo-distal scan length was 12 cm. A “small” FOV (diameter of 9.94 cm) was used for the ankle, and a “double small” FOV (diameter of 9.28 cm) for the wrist. All MDCT volumetric acquisitions were completed in a single tube rotation of 360° without table incrementation (no pitch).

Three **study protocols** were measured both in the left ankle and the right wrist phantom:

1. **CBCT**, study protocol
2. **MDCT (routine)**, protocol used in routine imaging at the authors’ institution
3. **MDCT (CTDI equivalent)**, protocol adapted to match the CBCT’s CT dose index volume (CTDIvol) values

Each of these three study protocols consisted of **three exposure levels**:

- “**low**”, suitable for pre-schoolers younger than 6 years
- “**medium**”, suitable for schoolers aged 6 to 12 years
- “**high**”, suitable for teenagers older than 12 years

In CBCT, the “high” protocol corresponded to the manufacturer-lowered pre-set for children. Prior to the dosimetric analyses, the study authors consensually agreed on the two other lowered CBCT exposure settings “medium” and “low”, with the aim of a barely sufficient image quality to securely make a diagnosis. The settings were based on initial experiences with phantoms, animal cadavers, and patients. The first MDCT protocol set (MDCT routine) consisted of exposure settings actually used in clinical routine at the authors’ institution. The second set [MDCT (CTDI equivalent)] was adapted to match the CTDIvol (16 cm phantom) values of the respective CBCT protocol, taking the underlying diverging CTDIvol phantoms in both machines into account. This exposure set was meant to show dose conformity between the modalities. All described protocols and exposure settings are summarized in Table 1.

**Table 1:** Exposure parameters for CBCT and MDCT protocols and phantoms.

Protocol	Phantom	Exposure level	kVp	mA	Rotation time (sec)	mAs (effective)	CTDIvol (16cm phantom)	DLP (mGy*cm)
CBCT	Ankle	low	80.0	4.0	6.000	24.0	1.1	14.5
		medium	84.0	6.0	6.000	36.0	2.1	26.9
		high	92.0	8.0	6.000	48.0	4.0	51.6
	Wrist	low	80.0	2.0	6.000	12.0	0.6	7.2
		medium	84.0	4.0	6.000	24.0	1.4	18.0
		high	88.0	6.0	6.000	36.0	2.5	32.5
MDCT (routine)	Ankle	low	100.0	30.0	0.500	15.0	1.6	20.3
		medium	120.0	30.0	0.500	15.0	2.5	29.5
		high	120.0	40.0	0.500	20.0	3.2	39.4
	Wrist	low	100.0	30.0	0.500	15.0	1.4	16.7
		medium	120.0	20.0	0.500	10.0	1.6	17.6
		high	120.0	30.0	0.500	15.0	2.2	26.3
MDCT (CTDI equivalent)	Ankle	low	80.0	20.0	1.000	20.0	1.1	13.5
		medium	100.0	50.0	0.375	18.8	2.1	25.3
		high	120.0	30.0	0.750	22.5	4.0	47.8
	Wrist	low	80.0	30.0	0.375	11.3	0.6	7.7
		medium	100.0	20.0	0.625	12.5	1.4	17.4
		high	120.0	40.0	0.375	15.0	2.5	29.6

Exposure settings, CTDIvol values and dose-length products (DLP) are shown for CBCT and MDCT in both extremity phantoms and for all examined imaging protocols.

**Fig 2:**
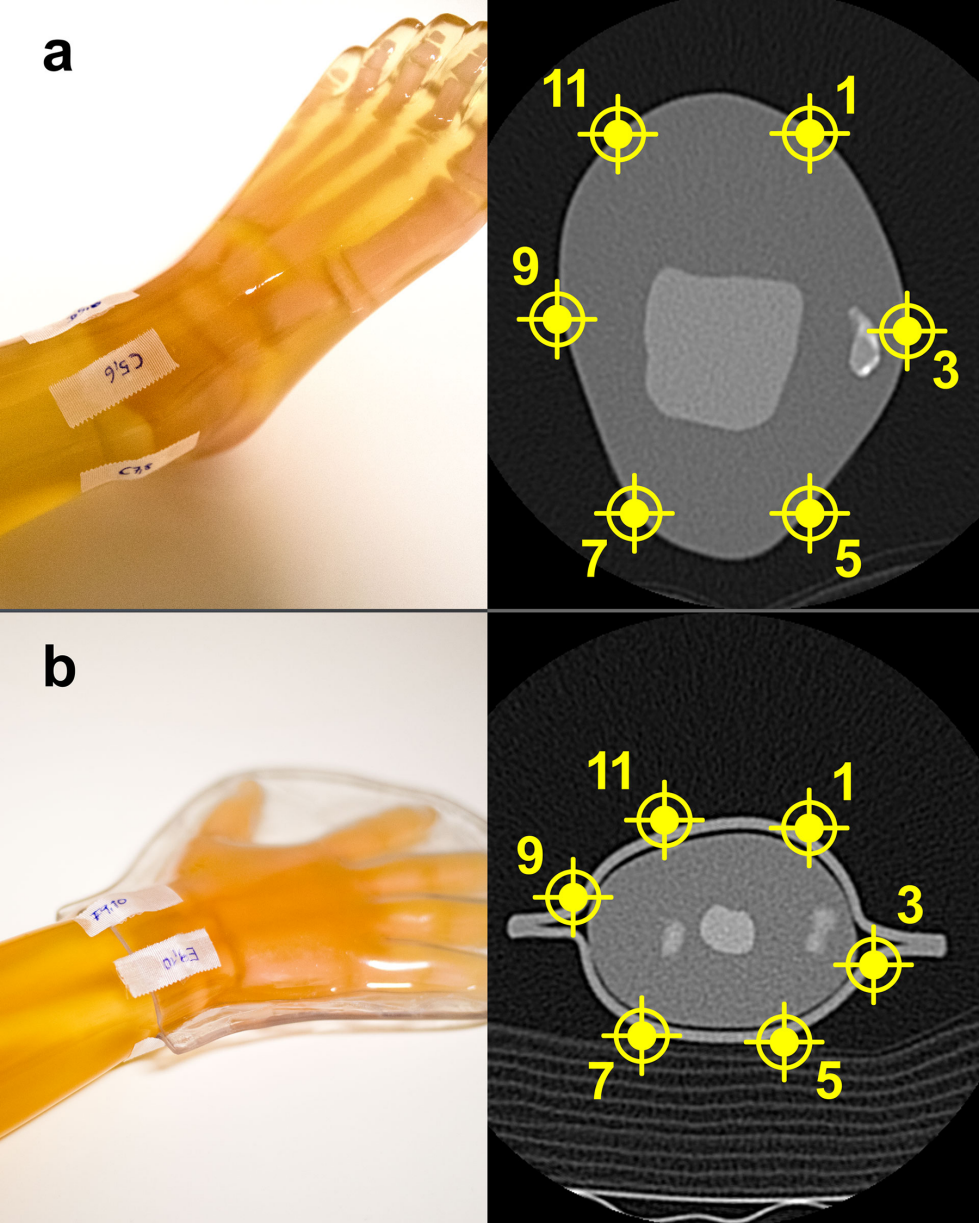
Positioning of TLDs around the ankle and wrist. Dosimeter pairs were attached to the phantoms in clockwise position at 1, 3, 5, 7, 9, and 11 o’clock as displayed. a) ankle phantom. b) wrist phantom.

Lithium fluoride (LiF) thermoluminescence dosimeters TLD-100^™^ square rods with diameters of 1×6 mm (Thermo Fisher Scientific Inc., Waltham, Massachusetts, USA) were used. TLDs were calibrated to the X-ray quality of both devices (CBCT and MDCT) individually. Dose results are based on calibration and correction factors determined at the Competence Center of Medical Physics and Radiation Protection, University Hospital of Graz, Austria. TLD glow-curves were read out on a Harshaw TLD Model 5500 reader with planchet heating system and WinREMS readout software (Thermo Fisher Scientific Inc., Waltham, Massachusetts, USA). Surface doses were measured in 6 circular positions around the examined joints. For each measurement a pair of TLDs was attached in the same way at every 1, 3, 5, 7, 9, and 11 o’clock position, seen in the direction of the extremity as shown in Fig 2. All dosimeters were consecutively irradiated 10 times per study protocol in order to ensure that a sufficient amount of radiation had been applied. Additionally, all protocols were repeated 3 times to prove the measurement test-retest reliability. After exposure TLDs were read out by a medical physicist with longstanding experience. A total of 322 (of 324) successful TLD readouts were performed, while 2 TLD readouts failed due to material fatigue.

After the respective image acquisitions, axial slices with thicknesses of 1.4 millimeters were reconstructed relative to the examined body part. The reconstruction kernel was “Standard” without iterative reconstruction in CBCT, and FC30 “Bone sharp” plus “AIDR” iterative reconstruction in MDCT.

FIJI 1.49v [20], an ImageJ distribution (open source image processing software, http://rsbweb.nih.gov/ij/) was used to measure mean pixel values and noise of air, cancellous bone, and soft tissue. Ellipsoid regions of interest (ROI) were placed in matching axial slices and positions and the resulting Hounsfield units (HU) were assessed. These measurements were repeated in 10 different matching image positions across all studies. Maximum and mean HU values of cortical bone and the respective standard deviations were read out in all series too. This information was used to calculate a normalization factor (NF = maximum cortical bone intensity of single study / maximum cortical bone intensity of all studies) applied to contrast-to-noise ratios (CNR), with the aim to extenuate device specific effects of differences in HU display [28, 29], and of various kVp settings [30, 31]. NF was 1.0 for the study containing the highest maximum cortical bone pixel value, and lower for all other exposure levels (compare S2 Dataset, listing all objective and subjective image measurements). Combined mean image noise (of air, cancellous bone, and soft tissue), normalized CNR [(mean cortical bone – mean air) / SD air * NF] and signal-to-noise ratios (SNR = mean [material] / SD [material]) were calculated for every CBCT and MDCT protocol as parameters for objective image quality.

Subjective image quality was rated consensually on a five grade Likert scale (1 = excellent, 2 = good, 3 = average, 4 = fair, 5 = poor) by three radiologists with 4, 6 and 28 years of experience in musculoskeletal CT. The assessed parameters included overall quality, contrast, sharpness, noise, beam hardening, aliasing, and ring artifacts.

Collected data was imported and analyzed in SPSS Statistics Version 21 software (IBM Corp., Armonk, NY). Descriptive statistics were used to explore the measured values. Mean values were compared with independent samples t-tests. Pearson correlations were used to assess relations between dose and image quality parameters. Intraclass correlation [two-way mixed average measures, ICC(3,k)] coefficients and a Bland-Altman plot were calculated to prove dose measurements’ test-retest reliability. P values less than 0.05 were assumed to be statistically significant.

An ethics committee approval was not needed for this phantom study.

## Results

In CBCT the mean surface dose was 3.0 ±1.9 mGy, averaged over low, medium, and high ankle and wrist protocols. Significantly higher surface doses were measured for MDCT (routine) with 3.9 ±1.1 (p<0.001). As expected, differences between the CBCT and the CTDI-matched MDCT (CTDI equivalent) protocol were not statistically significant (mean 3.0 ±1.9 mGy vs. 3.0 ±1.4 mGy, p = 0.903).

Significant linear correlations between surface doses and both CTDIvol (r = 0.957, p<0.001) and DLP (0.950, p<0.001) were found. Surface doses in CBCT were significantly lower compared to MDCT (routine) in the majority of the exposure levels apart from “high” in general (p = 0.633), “high” in the ankle phantom (p = 0.131), and overall in the ankle phantom (p = 0.053). Respective findings are summarized in Table 2.

**Table 2:** Independent samples t-test results between CBCT and MDCT (routine).

Phantom	Exposure level	Protocol	Valid pairs (n)	Mean (mGy)	SD (mGy)	Significance (p)
Ankle + Wrist	low + medium + high	CBCT	107	3.0	1.9	<0.001
		MDCT (routine)	108	3.9	1.2	
Ankle	low + medium + high	CBCT	53	3.8	2.1	0.053 ns
		MDCT (routine)	54	4.5	1.3	
Wrist	low + medium + high	CBCT	54	2.2	1.3	<0.001
		MDCT (routine)	54	3.4	0.7	
Ankle + Wrist	low	CBCT	36	1.3	0.5	<0.001
		MDCT (routine)	36	3.0	0.3	
	medium	CBCT	36	2.8	0.9	<0.001
		MDCT (routine)	36	3.7	0.8	
	high	CBCT	35	5.0	1.7	0.633 ns
		MDCT (routine)	36	5.1	0.9	
Ankle	low	CBCT	18	1.8	0.4	<0.001
		MDCT (routine)	18	3.0	0.4	
	medium	CBCT	18	3.4	0.8	<0.001
		MDCT (routine)	18	4.5	0.4	
	high	CBCT	17	6.4	1.2	0.131 ns
		MDCT (routine)	18	5.9	0.6	
Wrist	low	CBCT	18	0.9	0.2	<0.001
		MDCT (routine)	18	2.9	0.3	
	medium	CBCT	18	2.1	0.3	<0.001
		MDCT (routine)	18	3.0	0.3	
	high	CBCT	18	3.6	0.9	0.004
		MDCT (routine)	18	4.4	0.4	

Summarized results of independent samples t-tests in both phantoms and the three exposure levels for CBCT and MDCT (routine), including combinations among each other’s. Non-significant differences are indicated by the letters “ns”.

**Fig 3:**
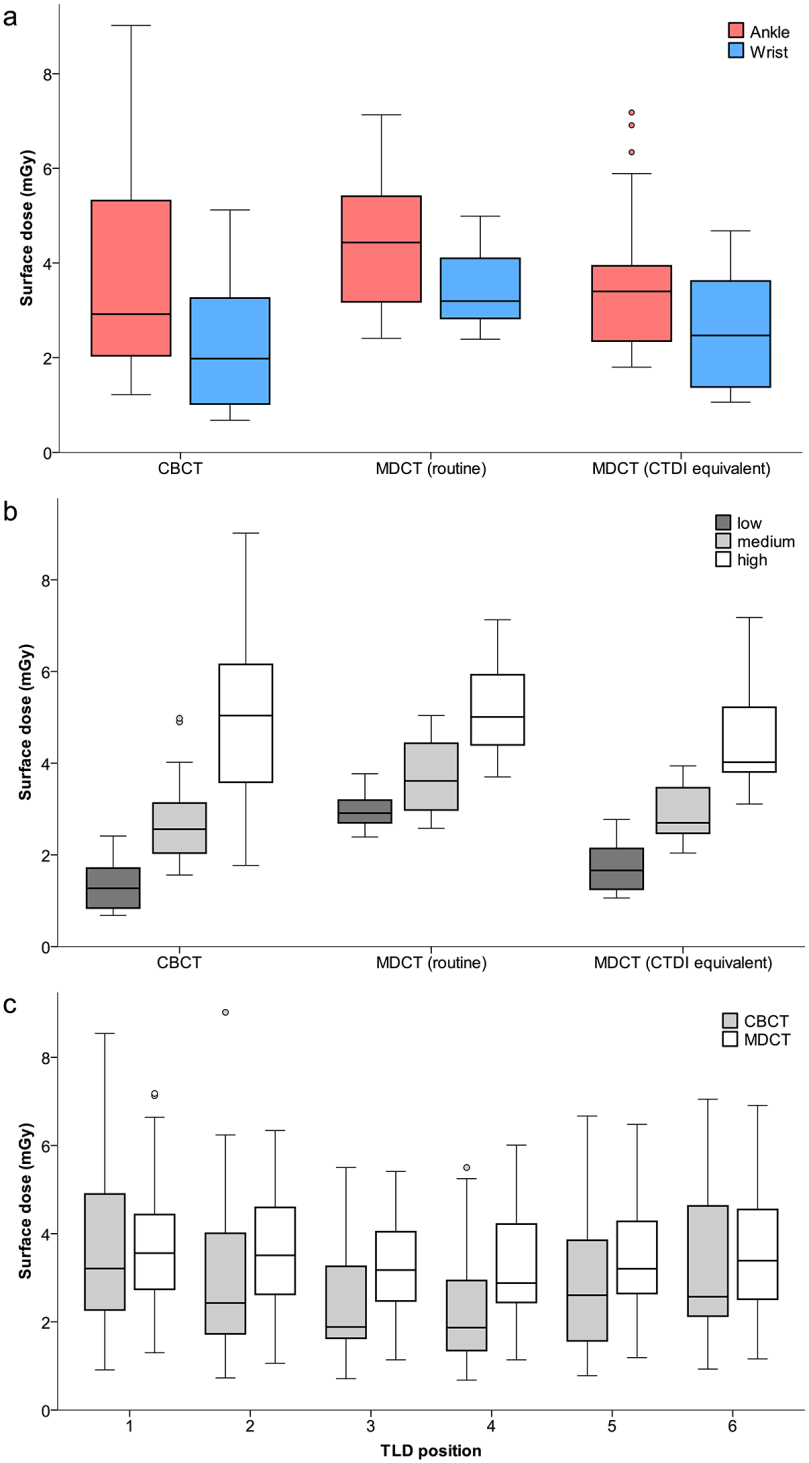
Box plots displaying surface doses in mGy for both examined devices. a) ankle and wrist, and b) for the different exposure settings “low”, “medium”, and “high”. c) Mean surface doses at different positions around the joints in CBCT and MDCT.

In CBCT the posterior TLDs at 5 and 7 o’clock positions showed significantly lower surface doses than the anterior positions at 1 and 11 o’clock (mean 2.5 ±1.5 mGy vs. mean 3.5 ±2.0 mGy, p = 0.014), accounted for by the incomplete CBCT tube rotation of 210 degrees. This is graphically depicted in Fig 3c. In MDCT, no significant differences between the surface doses at the different dosimeter positions were detected. Mean values of all measured surface doses are listed in Table 3; respective raw data is available in S1 Dataset.

**Table 3:** Measured surface doses at the six positions around the ankle and wrist.

Protocol	Phantom	Exposure level	TLD 1 mean (mGy)	TLD 2 mean (mGy)	TLD 3 mean (mGy)	TLD 4 mean (mGy)	TLD 5 mean (mGy)	TLD 6 mean (mGy)	Total TLD mean (mGy)	Total TLD SD (mGy)
CBCT	Ankle	low	2.2	1.7	1.6	1.4	1.6	2.1	**1.8**	**0.4**
		medium	4.3	3.4	2.6	2.7	3.1	4.3	**3.4**	**0.8**
		high	7.7	7.1	5.3	5.3	6.2	7.0	**6.4**	**1.2**
	Wrist	low	1.0	0.9	0.8	0.7	0.8	1.1	**0.9**	**0.2**
		medium	2.6	2.0	1.8	1.7	2.2	2.4	**2.1**	**0.3**
		high	4.4	3.7	2.8	2.8	3.9	4.3	**3.6**	**0.9**
MDCT (routine)	Ankle	low	3.2	3.3	2.9	2.7	2.9	3.0	**3.0**	**0.4**
		medium	4.3	4.5	4.3	4.3	4.4	4.9	**4.5**	**0.4**
		high	6.6	5.5	5.2	5.5	6.1	6.3	**5.9**	**0.6**
	Wrist	low	3.3	2.8	2.6	2.7	3.0	3.1	**2.9**	**0.3**
		medium	3.1	3.3	3.1	2.7	2.8	3.0	**3.0**	**0.3**
		high	4.6	4.7	4.3	4.2	4.3	4.1	**4.4**	**0.3**
MDCT (CTDI equivalent)	Ankle	low	2.4	2.4	2.0	1.8	2.1	2.3	**2.2**	**0.3**
		medium	3.4	3.5	3.2	3.0	3.3	3.7	**3.4**	**0.4**
		high	5.6	5.3	4.5	4.6	5.1	6.4	**5.2**	**1.1**
	Wrist	low	1.4	1.3	1.3	1.2	1.2	1.3	**1.3**	**0.1**
		medium	2.6	2.4	2.3	2.2	2.5	2.4	**2.4**	**0.2**
		high	4.1	3.9	3.7	3.8	3.9	4.0	**3.9**	**0.4**

Summary of dose measurements in mGy for both devices, phantoms and all used protocol settings. Mean values are displayed for each separate position and in total.

**Fig 4:**
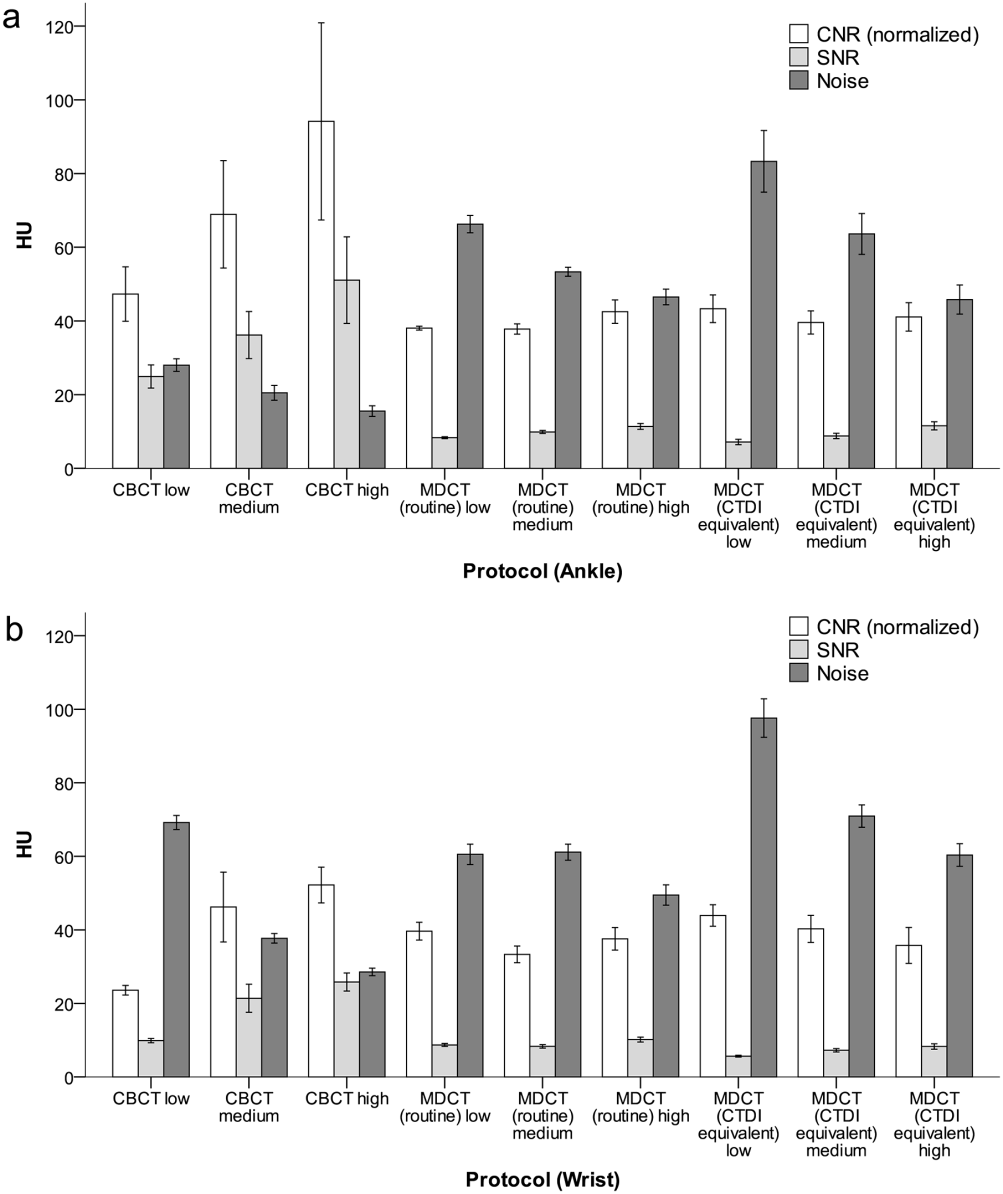
Objective image quality assessment. CNR, SNR and noise in a) ankle and b) wrist phantom across all exposure settings.

The objectively measured image parameters were significantly in favor of CBCT (all p<0.001), even though the noise reduction algorithm of iterative reconstruction had been enabled in MDCT: Mean noise was 33.3 ±17.8 HU in CBCT and 63.2 ±15.6 HU in MDCT. Normalized CNR was 55.4 ±28.5 HU in CBCT, and 39.4 ±5.1 HU in MDCT. SNR was 28.2 ±15.1 HU in CBCT and 8.8 ±1.8 HU in MDCT. Noise, CNR and SNR are graphically depicted in Fig 4.

**Fig 5:**
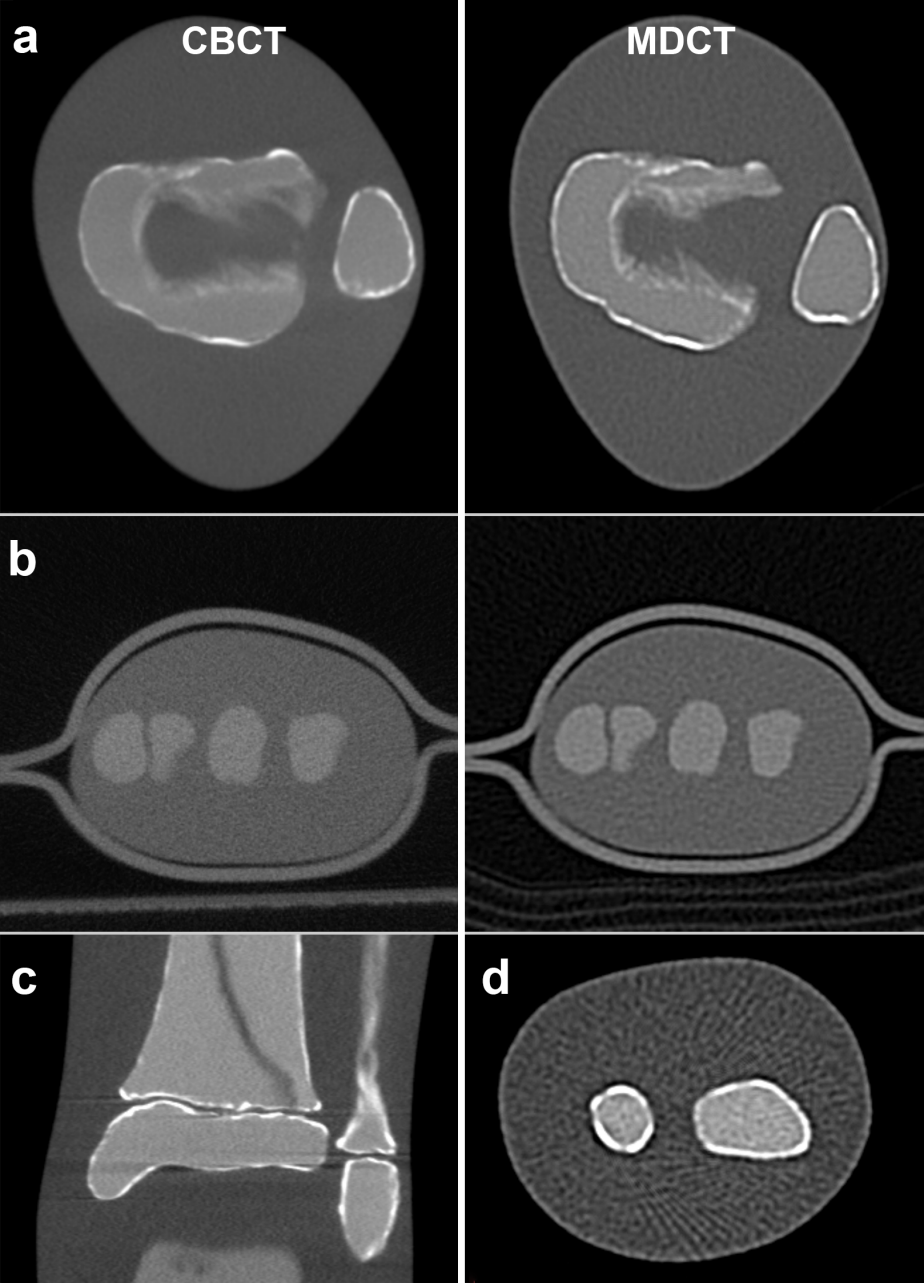
Examples of images quality at different dose levels and image artifacts. a) Side-by-side comparison of matching slices in CBCT (left) and MDCT (right) ankle examinations rated with good image quality. b) Wrist phantom scanned in CBCT (left) and MDCT (right) rated poor image quality. c) Beam hardening artifacts in CBCT. d) Aliasing artifacts and image noise in MDCT.

Subjective overall image quality decreased with lower exposure settings (R = -0.594, p = 0.009), and showed significant negative linear correlations with surface dose in both devices (R = -0.816, p = 0.048 in CBCT; and R = -0.693, p = 0.012 in MDCT). Overall image quality was not significantly different between both devices (CBCT 2.7 ±0.8 and MDCT 3.0 ±1.0 points, p = 0.456) and the study protocols [CBCT 2.7 ±0.8 and MDCT (routine) 2.3 ±0.5 points, p = 0.421]; neither was sharpness or contrast. Objectively measured and subjectively rated noise were significantly correlating (R = 0.809, p<0.001). Comparative examples of CBCT and MDCT images are given in Fig 5a and 5b. In contrast to CBCT, the raters did not detect any beam hardening artifacts in MDCT (example depicted in Fig 5c). On the other hand aliasing and noise were rated stronger in MDCT (p<0.001), exemplarily shown in Fig 5d. All subjective image ratings are listed in Table 4.

**Table 4:** Subjective image quality ratings.

Protocol	Phantom	Exposure level	Overall quality	Contrast	Sharpness	Noise	Beam hardening	Aliasing
CBCT	Ankle	low	3	1	3	2	3	2
		medium	2	2	2	2	3	1
		high	2	2	1	1	2	1
	Wrist	low	4	3	4	2	4	2
		medium	3	1	2	1	3	2
		high	2	1	1	1	3	2
MDCT (routine)	Ankle	low	3	1	3	4	1	4
		medium	2	2	3	3	1	3
		high	2	2	2	2	1	3
	Wrist	low	3	1	2	4	1	3
		medium	2	1	3	2	1	3
		high	2	1	2	3	1	4
MDCT (CTDI equivalent)	Ankle	low	4	1	4	5	1	4
		medium	4	2	4	4	1	3
		high	3	3	3	3	1	3
	Wrist	low	5	1	5	5	1	3
		medium	3	2	4	3	1	3
		high	3	2	3	3	1	3

Consensus rating of image quality listed for all study protocols and exposure settings. Ratings range from 1 = excellent to 5 = poor on a five point Likert scale.

**Fig 6:**
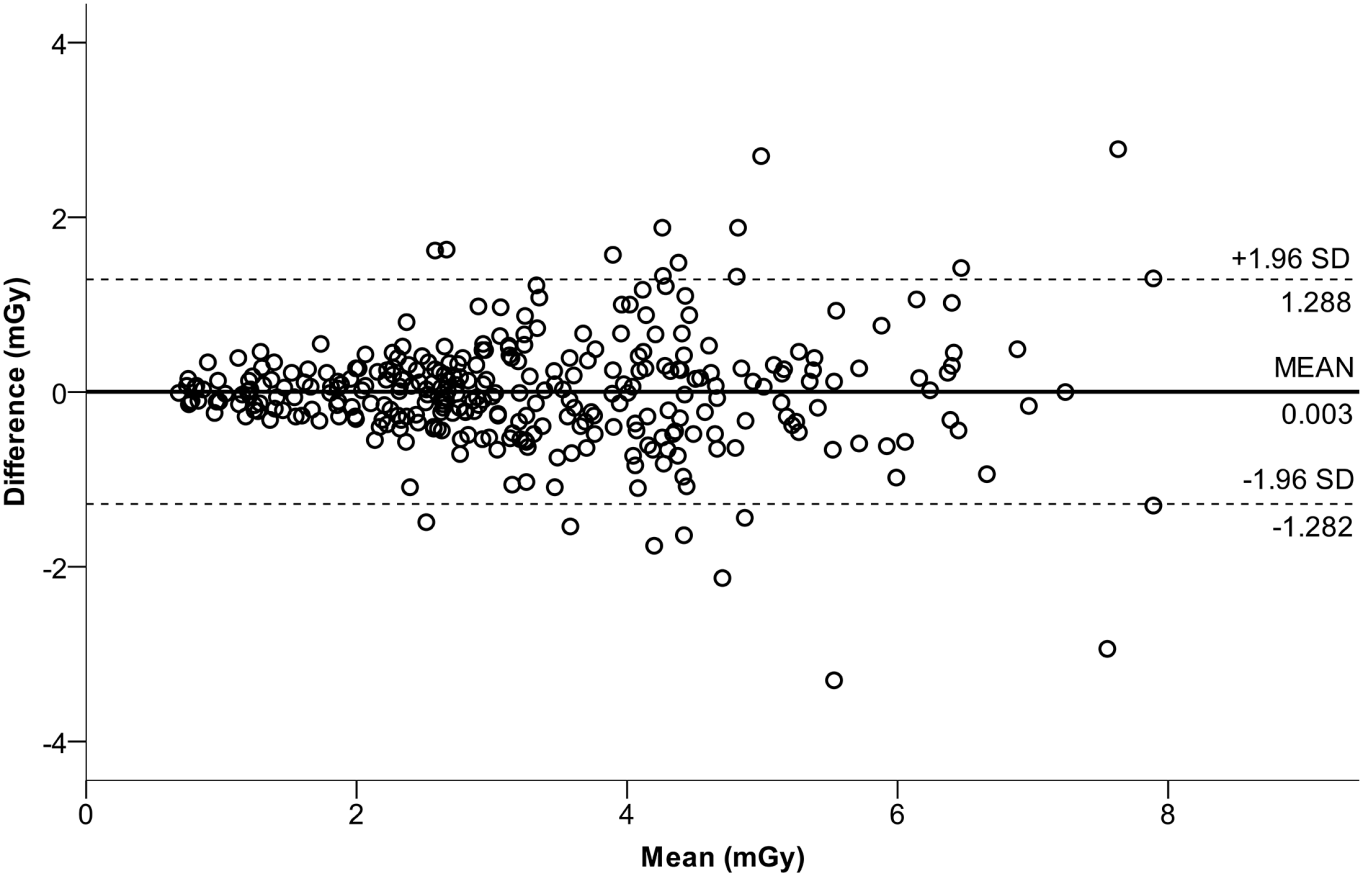
A Bland-Altman plot shows test-retest reliability of all TLD surface dose measurements among each other. Mean 0.003 (SD ±0.66) mGy shown as solid horizontal line, and upper and lower 1.96 SD intervals presented as dotted horizontal lines at 1.288 and -1.282 mGy respectively. All valid measurements from all six TLD positions were included in the plot.

Excellent test-retest reliability was shown for the TLD surface dose measurements by an overall ICC(3,k) of 0.953 (p<0.001). Split up between the modalities, the ICC was 0.946 (p<0.001) in CBCT and 0.922 (p<0.001) in MDCT. The measurement repeatability is depicted in Fig 6.

## Discussion

In this study surface doses applied by an extremity CBCT and a MDCT scanner in pediatric ankle and wrist phantoms were assessed. Adapted pediatric imaging protocols with age-related exposure settings were used to compare both scanners as realistically as possible.

To the best of our knowledge, this is the first study assessing and systematically comparing extremity CBCT doses in pediatric ankle and wrist phantoms. In adults, exceptional dose-saving potentials of CBCT compared to MDCT have been published for the knee and ankle region by Koivisto et al. [24, 25]. In contrast, recent studies by Lang et al. and Neubauer et al. did not confirm these findings and the authors reported comparable doses of extremity CBCT and MDCT examinations, when taking image quality into account [20, 22]. The currently available clinical pediatric extremity CBCT studies report doses that usually are lower than MDCT [23, 32]. As a result, dose optimization and possible dose savings still remain subject of debate and warrant future studies, especially in the radio-susceptible pediatric population.

In our study, we explicitly did not try to assess effective radiation doses, because different factors and unknown variables influence their correct calculation in pediatric extremities [33]. Mainly, the distribution of radiosensitive red bone marrow and relatively radio-insensitive yellow bone marrow are known to vary at different ages and to decrease until adulthood [34–36]. Additionally, inter-individual variety of marrow distribution and conversion may occur [37], rendering it impossible to predict the actual individual patient risk of an examination expressed by a specific effective dose value. Moreover, effective dose is believed not to be able to serve as a valid parameter of patient risk per se [38–41], and stochastic radiation induced damage may be underestimated [42, 43]. Effective dose was not intended to be applied to patients or children [38, 44], and appropriate pediatric tissue weighting factors remain a subject of ongoing debate and research [45–47]. In respect of the stated shortcomings in regard to effective dose in children, we have chosen to compare both modalities based on surface doses only.

In the current study image noise, normalized CNR, and SNR were examined as three basic, objectively measurable image quality parameters. In both CBCT and MDCT altering dose settings directly influenced the resulting image quality, as expected. Due to the missing pre-detector collimation in CBCT, scatter radiation additionally decreased the image quality through image artifacts [20, 22, 48, 49]. Therefore, scatter radiation is normally corrected by mathematical algorithms [50, 51], which may not equally correct for scatter induced artifacts in every situation. On the other hand, iterative reconstruction algorithms of the MDCT decrease image noise and alter the image impression [52, 53], which was not available or applied in CBCT. Due to the reasons given above, a direct comparison between different CBCT devices or CBCT and MDCT machines is further complicated regarding objective, and especially subjective image quality. We found quantitative image quality in favor of CBCT at comparative dose levels, whereas subjectively no significant difference was noted. However, alike Neubauer et al. and Lang et al. [20, 22] we detected more image-degrading beam hardening artifacts in CBCT.

In contrast to previous related studies by Koivisto et al. [24, 25] we have chosen TLDs over MOSFET dosimeters. The main reason was the better availability of, and longer experience with TLDs in our institution. Prior studies have not shown significant differences between both methods of dose measurements [54–56].

The following study limitations need to be mentioned: The most important limitation is the fact that phantoms were used instead of pediatric patients or cadaveric specimens, which we had favored due to reasons of availability, longevity and consistency. These phantoms of the ankle and wrist may not be completely anthropomorphic with different material compositions and X-ray absorption. Another important limitation is the choice of appropriate and matchable exposure settings. The general differences between the modalities complicate their comparison. The exposure parameters in the analyzed MDCT had been optimized and validated in clinical routine for many years, and this amount of dose titration was certainly not possible in the CBCT machine. We therefore decided to run two sets of protocols in MDCT, one actually used in clinical routine, and one that provided matching CTDIvol values with the CBCT scanner.

## Conclusions

Compared to an optimized MDCT, CBCT surface doses were significantly lower in a majority of the examined study protocols with superior objective, but only equivalent subjective image quality. Even though the previously reported extraordinary CBCT dose savings could not be substantiated in the examined pediatric ankle and wrist phantoms, the study results raise hope that CBCT could be a valuable low-dose alternative in pediatric extremity trauma imaging.

## Supporting information

S1 Dataset Raw data of surface dose measurements. (XLSX)

S2 Dataset Raw data of objective and subjective image quality measurements. (XLSX)

## Abbreviations

CBCT: Cone beam computed tomography
CNR: Contrast-to-noise ratio
CTDIvol: Computed tomography dose index volume
DLP: Dose length product
FOV: Field of view
HU: Hounsfield unit
ICC: Intraclass correlation coefficient
LiF: Lithium fluoride
MDCT: Multidetector computed tomography
NF: Normalization factor
SD: Standard deviation
SNR: Signal-to-noise ratio
TLD: Thermoluminescence dosimeter
